# Association Between Irritable Bowel Syndrome and Risk of Parkinson's Disease: A Systematic Review and Meta-Analysis

**DOI:** 10.3389/fneur.2021.720958

**Published:** 2021-09-22

**Authors:** XinYue Zhang, Zhen Svn, MengSi Liv, Yan Yang, Rui Zeng, Qian Huang, Qin Sun

**Affiliations:** ^1^College of Integrated Chinese and Western Medicine, Affiliated Hospital of Traditional Chinese Medicine, Southwest Medical University, Luzhou, China; ^2^Hengyang Medical School, University of South China, Hengyang, China; ^3^Drug Research Center of Integrated Traditional Chinese and Western Medicine, Affiliated Traditional Chinese Medicine Hospital, Southwest Medical University, Luzhou, China

**Keywords:** Parkinson disease, irritable bowel syndrome, co-occurrence, systematic review, meta-analysis

## Abstract

**Background:** Parkinson's disease (PD) and irritable bowel syndrome (IBS) are respectively one of the most common neurodegenerative diseases and functional bowel diseases in the world. Recent studies suggest that patients with IBS seem to have a higher risk of PD, which conflicts with the result of previous meta-analysis. Therefore, the purpose of this systematic review is to evaluate all available evidence, in order to clarify the association between PD and IBS.

**Methods:** Two reviewers independently searched the PubMed, Embase, Web of Science, and Cochrane library on April 25, 2021 to identify all records that explore the association between IBS and PD. All reports that clearly define PD and IBS and analyze the relationship between the two were included. The Newcastle-Ottawa scale was used to assess the risk of bias of included studies.

**Results:** Five studies from four articles involving 2,044,110 subjects were included in this analysis. The pooled results demonstrated a significant association between PD and IBS (1.48; 95% CI: 1.35–1.62, *P* < 0.001), with subtle heterogeneity (*I*^2^ = 0.0%, *p* = 0.585). The association was observed across genders and increased with age. However, the available evidence cannot allow a reliable analysis of the causal relationship between IBS and PD.

**Conclusion:** This study demonstrates a higher risk of PD among subjects with IBS. Future studies are required to further clarify the causation and underlying mechanism of the association.

## Introduction

Parkinson's disease (PD) is the second most common neurodegenerative disease in the world after Alzheimer's disease, which is characterized by static tremor, bradykinesia, and rigidity (1). One to two per 1000 of the population are affected by PD, and the incidence increases with age (2). Typical neuropathological features include the presence of Lewy bodies or Lewy neurites- containing aggregation of α-synuclein and the loss of dopaminergic neurons in the substantia nigra. The resultant depletion of dopamine in the basal ganglia leads to movement disorders (3). The exact mechanism of PD remains unclear, and studies indicate that it is the result of a complicated interplay between genetics and the environment. Some risk factors such as exposure to pesticides, traumatic brain injury, melanoma history have been confirmed (4–6). Due to the slowly progressive nature, PD often begins several years before diagnosis (7, 8). Some non-motor features such as dementia, gastrointestinal changes, and olfactory dysfunction are common in early PD and may be the precursor symptoms of PD, with more and more attention (9, 10). A growing number of studies have found significant associations between Parkinson's disease and multiple gastrointestinal symptoms that originate from changes in intestinal function and structure or in associated neural structures (11, 12). Furthermore, the gut microbiota and its relevant metabolites in PD have changed (13, 14). These findings support the gut-to-brain transmission hypothesis that the pathological process may spread from the gut to the brain (15, 16).

Irritable bowel syndrome (IBS) is one of the most common functional bowel disorders, afflicting more than 9% of adults worldwide according to Rome III criteria, and is characterized by changes in bowel habit associated with chronic or recurrent abdominal pain that is either relieved or worsened by defecation (17, 18). The underlying mechanisms of IBS include genetic factors, post infectious changes, altered gut microbiota, immunological alterations, low-grade mucosal inflammation, and changes in intestinal permeability (19). Its diagnosis requires the exclusion of known organic diseases with similar symptoms, such as inflammatory bowel disease, tumors, and coeliac disease (20). In recent years, the relationship between gut microbiota and irritable bowel syndrome has aroused significant interest in research and clinical fields. Some studies have found that gut microbiota may be a potential source and driving factor of clinical symptoms (21, 22). Furthermore, certain neurological symptoms unrelated to the gastrointestinal tract, such as anxiety, depression and mood disorders, are common in patients with IBS, which cannot be explained by health-care-seeking behavior alone (23–25). These associations support that defines IBS as a gut-brain disorder and that the pathophysiology of IBS may contribute to brain alterations.

PD and IBS may be related because the pathophysiology of both potentially involves the gut microbiota and the gut-brain axis. Previous studies have demonstrated that constipation is a prodromal symptom of PD (9). Considering the significant differences in definition and epidemiological characteristics between IBS and constipation, exploring the association between IBS and PD can help further identify populations at high risk for PD, provide new approaches for prevention, diagnosis, and treatment, and optimize policy-making. A previous meta-analysis by Fu et al. showed no statistical association between IBS and PD, but newly published studies based on a large population found that the association seems to exist (11, 26). Therefore, we conducted this meta-analysis and systematic review to determine whether IBS patients have a higher risk of PD.

## Materials and Methods

The present systematic review and meta-analysis followed the Preferred Reporting Items for Systematic Reviews and Meta-Analyses (PRISMA 2020) guidelines (27). The protocol for this study is not registered.

### Search Strategies

We systematically searched the PubMed, Embase, Web of Science, and Cochrane Libraries on April 25, 2021 using subject words combined with free words to identify all studies that investigate the correlation between IBS and PD, without any search filters or language restrictions. A manual search for additional relevant reports using the reference lists of included articles and related reviews was also performed.

(1) Subject words: irritable bowel syndrome; irritable colon. Free words: irritable bowel syndromes; syndrome, irritable bowel; mucous colitis; colon spasm; colonic diseases, functional; colonospasm; functional colonic diseases; irritable colon syndrome; mucomembranous colitis; spastic colitis; spastic colon; unstable colon.

(2) Subject word: Parkinson disease. Free words: idiopathic Parkinson disease; Lewy body Parkinson disease; Parkinson's disease; primary Parkinsonism; paralysis agitans; idiopathic Parkinsonism; Parkinson dementia complex; primary Parkinsonism.

The detailed search strategy in each database is available in Supplementary Material.

### Study Selection

Reports that met the following specific criteria would be considered for inclusion: (1) The study population was patients with PD or IBS. (2) In the case-control study, cases with PD were diagnosed according to standard clinical criteria or identified by the diagnostic code in patient medical records. Controls were healthy subjects or people without previous PD diagnosis from the same population. (3) The IBS cohort in the cohort study was diagnosed according to Rome criteria or questionnaire data, or identified by diagnostic code in patient medical records. The other cohort was from the same population without a history of IBS. (4) Study outcome reported the following measures of association: odds ratio (OR), relative risk (RR), or hazard ratio (HR) with corresponding 95% confidence interval (CI), or sufficient raw data were provided to calculate these ratios. (5) The study type was cross-sectional, case-control or cohort design.

Reports that meet any of the following criteria would be excluded: (1) studies not derived from new data, including meta-analyses, systematic reviews, reviews, letters, and comments. (2) Case reports or case series with fewer than 10 patients. (3) Conference abstracts that full-text were already published. (4) Reports with fewer cases in studies with overlapping data. (5) Single-arm studies lacking a control group. (6) Non-human studies, such as animal experiments.

Based on the inclusion and exclusion criteria described above, two reviewers independently reviewed the records initially retrieved. The exclusion reasons were recorded and cross-checked.

### Data Extraction and Quality Assessment

For eligible studies, two reviewers independently extracted the following data: first author, year of publication, region, age, sex, study period, study design, population source, number of cases and controls or different cohorts, diagnostic criteria of IBS and PD, adjusted confounding factors, follow-up time, and risk estimates with corresponding 95% CI.

The cohort design version and case-control design version of the Newcastle-Ottawa scale (NOS) were used to evaluate the included cohort studies and case-control studies, respectively, which evaluated the selection, comparability, and exposure (case-control study)/outcome (cohort study) (28). Report with a score of more than 6 was classified as high quality, otherwise as low quality.

Any discrepancy from study selection to quality assessment was resolved by consensus through discussion among all authors.

### Statistical Analysis

In this study, Stata/MP 16.0 was used for all data analyses. Adjusted risk estimates with corresponding 95% CI were used as the primary endpoints of interest for pooling. Only when a study did not report an adjusted risk estimate, the crude risk estimate would be extracted and annotated. Given that the incidence of PD is low, we assumed that OR and HR could be considered equally. So, all the risk estimates were treated as HR in our meta-analysis, which represents the relative risk of developing PD in IBS patients compared to controls. We used the inverse variance method of DerSimonian and Laird to pool the adjusted HRs, and *P*-value ≤ 0.05 was considered statistically significant (29). Cochran's *Q*-test and Higgins' *I*^2^ statistics were performed to assess heterogeneity between included studies. Sensitivity analysis was performed by excluding one study at a time and then repeating the analysis. Furthermore, we tested the robustness of the pooled results by comparing the results from random-effects and fixed-effect models. Low heterogeneity was considered when *I*^2^ <50% and *P* > 0.1, and the fixed-effects model was used. Inversely, the random-effects model was used, and the source of heterogeneity was analyzed by reviewing characteristics of included studies. Subgroup analysis was performed according to study design, gender, age, follow-up time, and study quality. We applied Begg's and Egger's tests to assess publication bias. When the *P*-values of both Begg's and Egger's tests ≥ 0.05, it was considered that there was no publication bias.

## Results

### Study Characteristics

**Figure 1:**
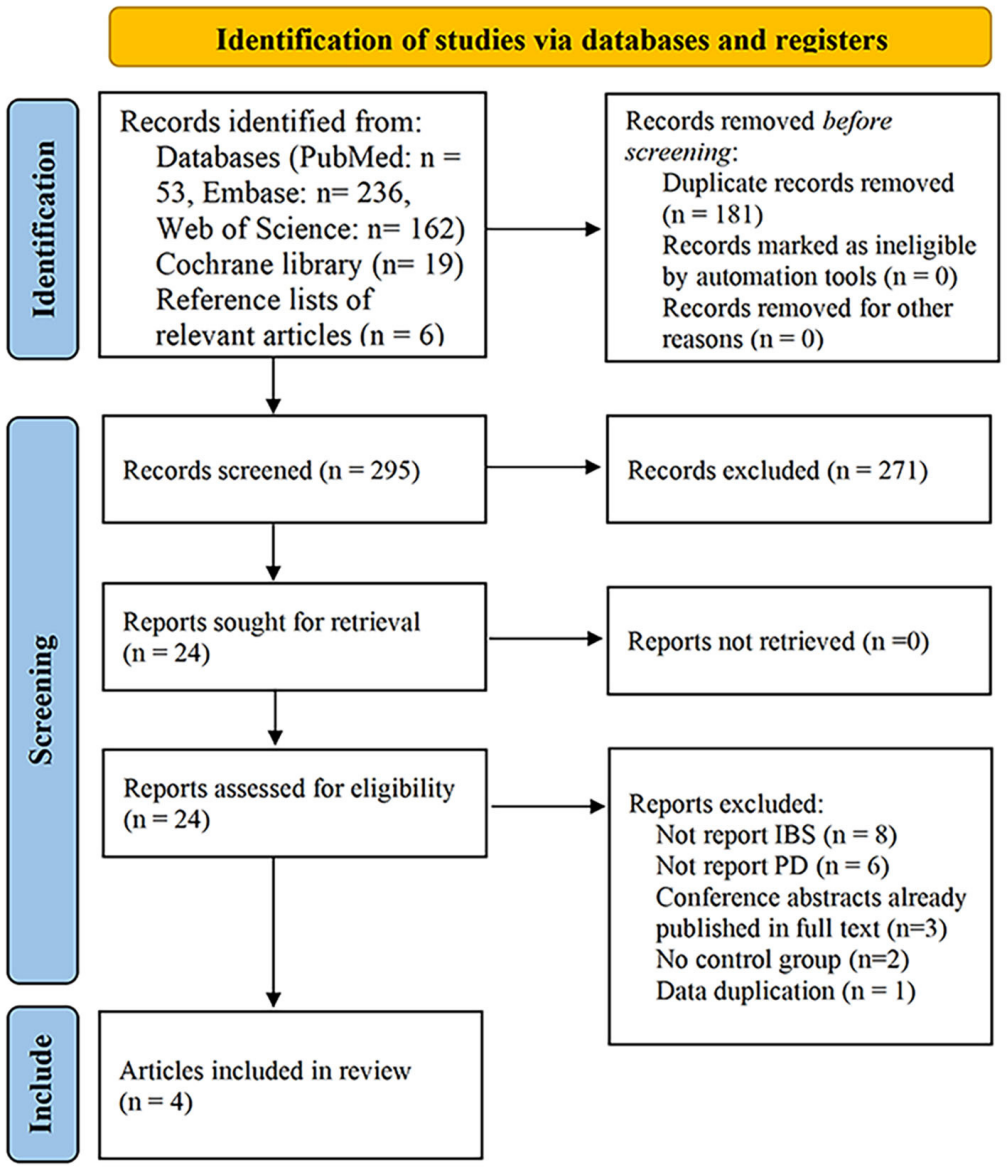
Flow diagram of the study select process.

The search strategy described above yielded 476 potential records, of which 53 came from PubMed, 236 from Embase, 162 from Web of Science, 19 from Cochrane library, and six from reference lists of relevant articles. After excluding duplicated records and screening titles and/or abstracts, 452 articles were excluded, as they were reviews, systematic reviews, study protocols, animal trials, letters, or comments. The remaining 24 reports were further assessed by a full-article review. Of these, eight did not report IBS, six did not report PD, three were conference abstracts that were already published in full, two did not have a control group, and cases in one (30) were included in one eligible study with a larger sample size. The list of articles excluded after a full-text reading is available in Supplementary Material. Finally, four studies involving 2,044,110 subjects were included (26, 31–33). Three of them were based on the nationwide or regional registers (26, 31, 32), and one was a case-control study based on patients recruited (33) (Figure 1).

Three large-scale studies were conducted in Taiwan region, Sweden, and Finland (26, 31, 32). Both the Taiwanese and Finnish study designs were cohort studies (26, 31). Sweden conducted nested case-control and cohort studies based on the Swedish Patient Register and Swedish Twin Registry, respectively (32). But the PD cases of both were identified from the Patient Register, and the number of PD patients included in the case-control study was more than that in the cohort study, that is, the cases in the cohort study were included in the case-control study (32). Therefore, we included only the Swedish case-control study in the meta-analysis, except for the subgroup analysis by the study design. Three studies based on national or regional databases each included more than 100,000 subjects, and all PD patients were diagnosed after the onset of IBS (26, 31, 32), while the case-control study conducted by Lee et al. recruited a relatively small group of 87 subjects and the sequence of PD and IBS diagnosis was unknown (33). Except for the cohort study in Sweden that used an algorithm involving self-reported abdominal symptoms based on the ROME II criteria to identify IBS cases (32), studies based on the nationwide or regional register all used the International Classification of Diseases codes to identify PD and IBS cases (26, 31, 32). Study conducted by Lee et al. used Korean Rome III and United Kingdom PD Society Brain Bank to diagnose patients with IBS and PD, respectively (33). The median or mean age of patients in all studies was over 50 years (26, 31–33). The Taiwanese and Finnish studies had a median follow-up of ~6 years (26, 31), the Swedish cohort had a median follow-up of 15.1 years (32), and the study by Lee et al. had a median follow-up of 46 months (33). The detailed information is shown in Table 1.

**Table 1:** Main characteristics of the studies included in this study.

**Author**	**Year**	**Study population**	**Study period**	**Study design**	**Gender (F/M)**	**PD identification**	**Cases**	**Comparators**	**IBS ascertainment**	**PD ascertainment**	**Adjustment**	**Median followed up-yr**.
Lai et al. (31)	2014	Taiwanese	2000-2010	Cohort	IBS+: 12,843/11,032; IBS-: 51,372/44,128	After	IBS+: 23,875	IBS-: 95,500	ICD code	ICD code	Gender, age, head injury, hypertension diabetes mellitus, hyperlipidemia, dementia, cerebrovascular disease, depression, and chronic kidney disease	IBS+: (mean, SD) 6.32 (3.12); IBS-: mean 6.22 (3.15);
Liu et al. (32)	2021	Swedish	1964-2010	Cohort; nested case-control;	Cohort: IBS+: 1,977/1,069; IBS-: 21,610/ 19,569 Case-control: PD+: 24,757/31,807; PD-: 742,710/ 954,210	After	Cohort: IBS+: 3,046; case-control: PD+: 56,564	Cohort: IBS-: 41,179; case-control: PD-: 1,696,920	ICD code	ICD code	Cohort: gender, age, highest achieved education, smoking history, alcohol consumption in the last month, and comorbidity index; case- control: gender, birth year, highest achieved education, country of birth, comorbidity index, chronic obstructive pulmonary disease;	Cohort: 16.0; case-control: NP;
Mertsalmi et al. (26)	2021	Finnish	1998-2014	Cohort	IBS+: 20,114/8,036; IBS-: 71,615/27,174	After	IBS+: 28,150	IBS-: 98,789	ICD code	ICD code	Age, gender, depression, anxiety disorders, and chronic obstructive pulmonary disease	6.3 (IQR 2.9–9.9)
Lee et al. (33)	2018	Korean	2013–2015	Case-control	PD+: 20/15, PD-: 26/26	Unspecific	PD+: 35	PD-: 52	Korean Rome III	UK PD Society Brain Bank	None	3.8 (2.6–4.3)

*IBS, irritable bowel syndrome; PD, Parkinson's disease; ICD, International Classification of Diseases; F/M, female/male; NP, not provided; IQR, inter quartile range*.

All cohort studies and case-control study based on nationwide or regional register had high quality with scores ranging from 8 to 9 (26, 31, 32). These studies were consistently of reasonable quality, with appropriate participants selection, clearly defined exposures, and adequate follow-up for outcomes. However, the Taiwanese and Finnish studies did not control detection bias (26, 31). The case-control study conducted by Lee et al. had an NOS score of six, mainly due to the potential selection bias (33) (Table 2).

**Table 2:** The quality assessment of included studies.

**Study (cohort)**	**Representativeness of exposed cohort**	**Selection of non-exposed cohort**	**Ascertainment of exposure**	**Outcome not present before study**	**Comparability**	**Assessment of outcome**	**Follow-up long enough**	**Adequacy of follow up**	**Quality score**
Lai 2014	⋆	⋆	⋆	⋆	⋆	⋆	⋆	⋆	8
Liu 2021	⋆	⋆	⋆	⋆	⋆	⋆	⋆	⋆	8
Mertsalmi 2021	⋆	⋆	⋆	⋆	⋆	⋆	⋆	⋆	8
**Study (case-control)**	**Case definition**	**Representativeness of the cases**	**Selection of Controls**	**Definition of Controls**	**Comparability**	**Ascertainment of exposure**	**Same method⋆**	**Non-Response rate**	**Quality score**
Liu 2021	⋆	⋆	⋆	⋆	**⋆⋆**	⋆	⋆	⋆	9
Lee 2018	⋆			⋆	⋆	⋆	⋆	⋆	6

### The Association Between IBS and Risk of PD

**Figure 2:**
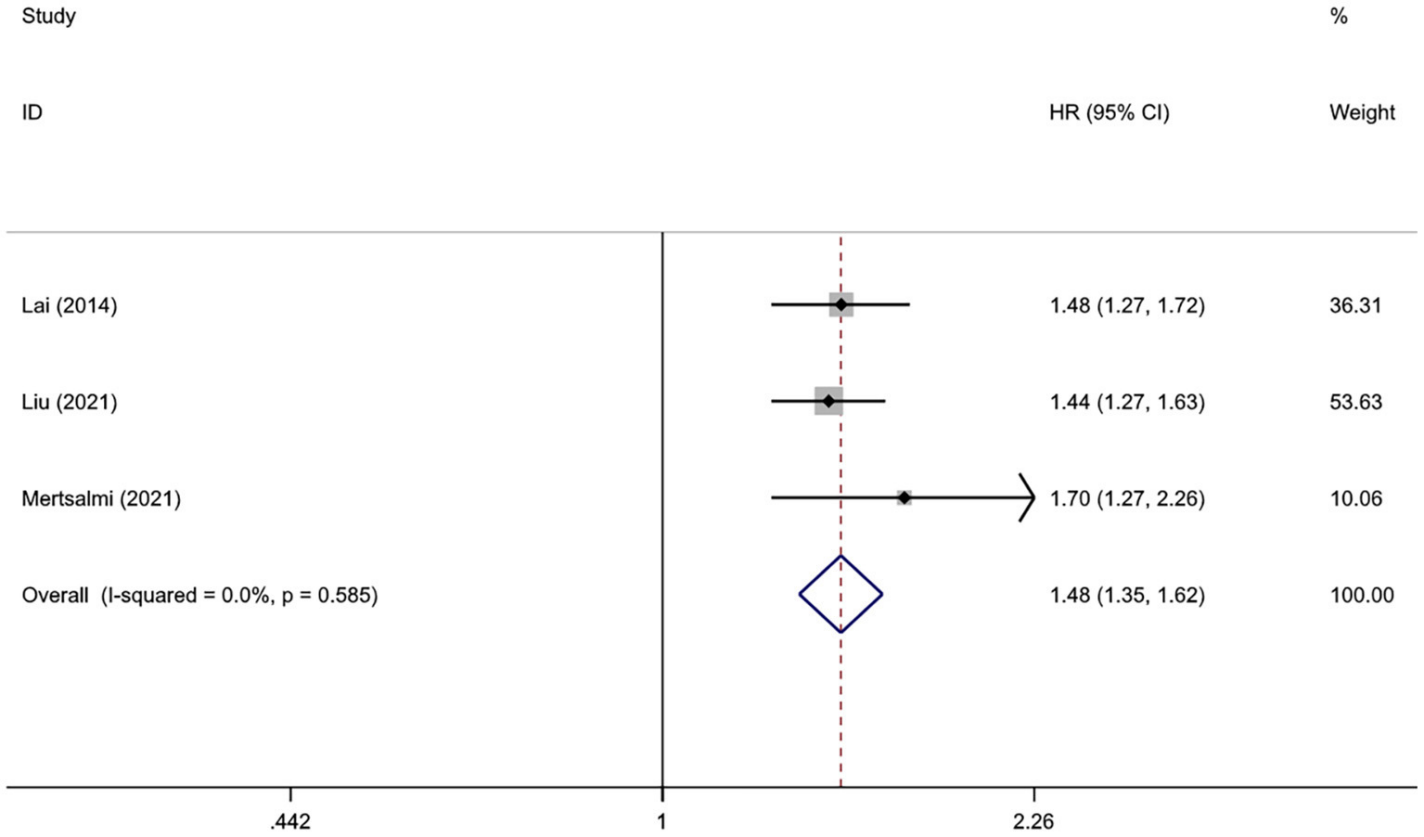
Forest plot of the associations between irritable bowel syndrome and risks of Parkinson's disease.

Considering the relatively small sample size and high risk of bias in the Korean study, we removed this study from the overall quantitative meta-analysis and included it only for subsequent qualitative and subgroup analyses. The pooled result of three studies involving 1,999,798 subjects showed a significant association between IBS and PD (26, 31, 32). Compared with those without IBS, the HR of PD onset was 1.48 (95% CI: 1.35–1.62, *P* < 0.001) among patients with IBS. The heterogeneity in this analysis was insignificant (*I*^2^ = 0.0%, *P*_heterogeneity_ = 0.585) (Figure 2). All patients in this analysis were diagnosed with PD after IBS onset.

### Subgroup Analyses

We performed meta-analyses of subgroup based on the study design, gender, and study quality (Table 3). Since the available data of age and follow-up time did not support quantitative analysis, we conducted a qualitative evaluation for them as comprehensive as possible.

**Table 3:** Subgroup analysis of the association between IBS and PD risk.

**Subgroup**	**No. of studies**	**Hazard ratio**	**95% CI**	***P*_**overalleffect**_**	***I*^**2**^ static**	***P*_**heterogeneity**_**
**Gender**						
Male	3	1.45	1.25 to 1.67	<0.001	0.3%	0.367
Female	3	1.58	1.27 to 1.96	<0.001	62.3%	0.071
**Study design**						
Cohort	3	1.49	1.31 to 1.69	<0.001	0.0%	0.428
Case-control	2	1.43	1.26 to 1.62	<0.001	47.8%	0.166
**Study quality**						
High	3	1.48	1.35 to 1.62	<0.001	0.0%	0.585
Low	1	0.32	0.04 to 2.81	0.294	–	–

According to study design, pooled result of the cohort studies showed that the IBS cohort had a significantly higher risk of PD than the non-IBS cohort (HR = 1.49; 95% CI: 1.31–1.69), with subtle heterogeneity (*I*^2^ = 0.0%, *P*_heterogeneity_ = 0.428) (26, 31, 32). The pooled result of case-control studies showed that there was also a significant association between IBS and PD (HR = 1.43; 95% CI: 1.26–1.62), with acceptable heterogeneity (*I*^2^ = 47.8%, *P*_heterogeneity_ = 0.166) (32, 33).

In subgroup analysis according to gender, IBS was significantly associated with risk of PD in both male and female subjects, with pooled HRs of 1.45 (95% CI: 1.25–1.67, *I*^2^ = 0.3%, *P*_heterogeneity_ = 0.367) and 1.58 (95% CI: 1.27–1.96, *I*^2^ = 62.3%, *P*_heterogeneity_ = 0.071), respectively (26, 31, 32). The high heterogeneity may be due to the small sample bias in the Finnish study. After excluding the Finnish study, the heterogeneity of female subgroup disappeared (*I*^2^ = 0.0%, *P*_heterogeneity_ = 0.878), and the statistical significance still existed (HR = 1.43, 95% CI: 1.26–1.62).

According to the NOS score, excluding the study of Lee et al. to combine the results of low bias risk studies, the pooled HR was 1.48 (95% CI: 1.35–1.62), with subtle heterogeneity (*I*^2^ = 0.0%, *P*_heterogeneity_ = 0.585), indicating that the results from high-quality studies were consistent (26, 31, 32).

In terms of impacts of age, in the study based on the Taiwanese population, it was found that the HR of PD increased by 10% with age (per 1 year) (31). In the Swedish population, the risk of PD after IBS diagnosis tended to be higher in the elderly (≥50 years at the time of IBS diagnosis) than in young people (<50 years), although the interaction test was not statistically significant (*P* = 0.11) (32). In the Finnish population, the authors reported only that the results were similar to the main analysis in subjects aged at least 50 years at the time of IBS diagnosis, but data were not presented (26). The impact of age was not assessed in the Korean study (33).

Studies in Taiwan and Finland performed stratified analysis for the association between IBS and risk of PD according to the follow-up time that started from the IBS diagnosis until the occurrence of PD (26, 31). The Taiwanese study showed a significant association between IBS and PD in both groups with less than and over 2 years of follow-up, but the hazard of developing PD was higher during the early 2 years of follow-up (HR = 1.77; 95% CI: 1.33–2.36 vs. 1.38; 95% CI: 1.16–1.66, respectively) (31). In the Finnish population, the highest HR was observed during the first 2 years of follow-up (HR = 2.96; 95% CI: 1.78–4.92), the relative difference between the two cohorts diminished during the 2 to 5 years of follow-up (HR = 1.08; 95% CI: 0.61–1.93), and the HR demonstrated an increase at follow-up over 5 years (HR = 1.53; 95% CI: 0.98–2.38) although not statistically significant (26). Furthermore, in the Swedish population, stratified analysis was also conducted according to the interval between IBS diagnosis and PD diagnosis (32). A significant association between IBS and risk of PD was observed when follow-up was less than 5 years (HR = 1.31; 95% CI: 1.06–1.61), more than 5 years (HR = 1.53; 95% CI: 1.30–1.79), less than 10 years (HR = 1.47; 95% CI: 1.26–1.72), and more than 10 years (HR = 1.38; 95% CI: 1.11–1.71) (32). Similar to age, the impact of follow-up was not assessed in the Korean study (33).

### Evaluation for Publication Bias

The *P*-values of Begg's and Egger's tests were 1.00 and 0.59, respectively, indicating that there was no publication bias. Due to the small number of included studies, the funnel plot was not carried out.

### Sensitivity Analysis

**Figure 3:**
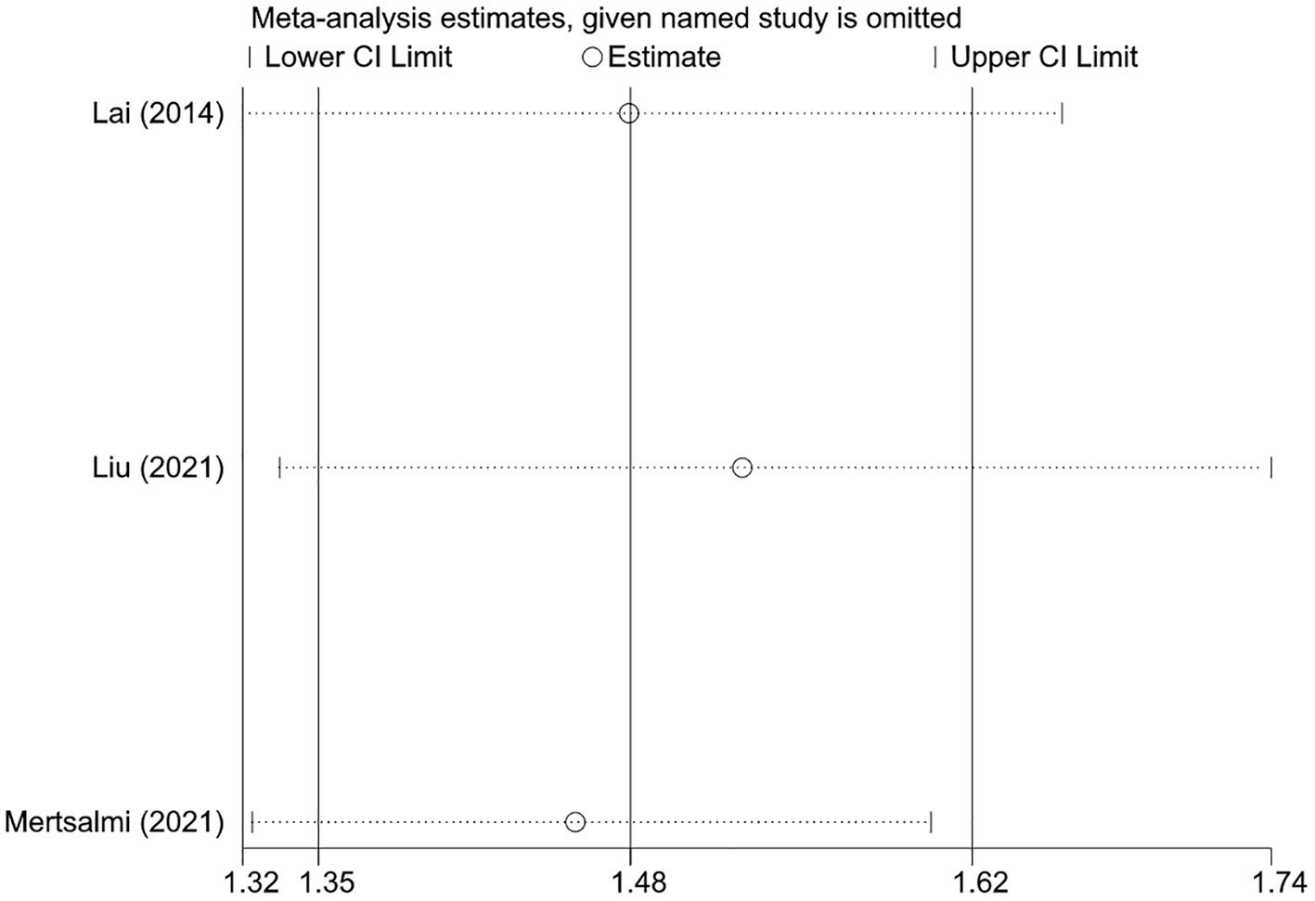
The sensitivity analysis of the associations between irritable bowel syndrome and risks of Parkinson's disease.

Sensitivity analysis was performed for pooled results by excluding one study at a time and using both random-effects and fixed-effects models. Results were stable and the conclusion was not changed when excluding one study at a time (Figure 3). Results from random and fixed-effects models were inconsistent in the case-control design subgroup. By reviewing the included studies, we found that the control subjects included in the study by Lee et al. came from patients without a history of neurological disorders who visited the gastroenterology outpatient at their institution, which may be a greater probability of IBS (33). Therefore, the pooled result of case-control studies using random-effects model that gives greater weight to the subjects included by Lee et al. may be unreliable due to the risk of patient selection bias. Except for this subgroup, the pooled results using different effects models were similar in other analyses (Table 4).

**Table 4:** Comparison of use of random-effects vs. fixed-effects models.

**Analysis groups**	**HR (95% CI), fixed-effects model**	**HR (95% CI), random-effects model**
Overall	1.48 (1.35–1.62)	1.48 (1.35–1.62)
Male group	1.45 (1.25–1.67)	1.45 (1.25–1.67)
Female group	1.50 (1.33–1.69)	1.58 (1.27–1.96)
Cohort design group	1.49 (1.31–1.69)	1.49 (1.31–1.69)
Case-control design group	1.43 (1.26–1.62)	1.00 (0.28–3.54)
High-quality group	1.48 (1.35–1.62)	1.48 (1.35–1.62)
Low-quality group	0.32 (0.04–2.81)	0.32 (0.04–2.81)

## Discussion

This is the first meta-analysis and systematic review that focuses on the association between IBS patients and risk of PD. Our study found a 48% increased risk of PD in subjects with IBS compared to those without IBS. There was little evidence of heterogeneity. Based on high-quality studies involving more than 2 million subjects, the results from this analysis can be considered a stringent and reliable estimate, despite the relatively small number of studies included.

Compared with the study of Fu et al. (11), we added two large nationwide sample studies from Sweden and Finland (26, 32), and excluded the case-control study of Mertsalmi et al. with patients that might overlap with the Swedish nationwide study (26, 30). In fact, the previous meta-analysis did not observe statistical association between IBS and PD mainly due to the study of Lee et al. (11, 33). The study by Lee et al. did not focus on exploring the association between IBS and PD and therefore may introduce a risk of bias in patient selection. In addition, due to the large heterogeneity, the random-effects model was used for their analysis, which resulted in a more conservative estimate (11). Due to the significantly increased data, the present meta-analysis obtained a more accurate prediction interval, and the sample size included was sufficient to demonstrate the association between IBS and risk of PD.

Subgroup analysis showed that significant increased risk of PD was observed in IBS subjects across genders. In the Finnish study, no higher risk of PD was observed in male IBS subjects, possibly due to a smaller sample size and therefore lack of statistical power (26). Majority of included studies suggested that the HR for PD increased with the age at IBS diagnosis (26, 31, 32).

Unlike the association between inflammatory bowel disease and PD (34), we found that the study design did not seem to be the source of heterogeneity in each analysis, and the results of the subgroup analyses did not differ when divided again according to case-control and cohort studies, which further demonstrated the stability of pooled results. In addition, the Swedish study conducted a sensitivity analysis adjusting for constipation, and the result showed that the association between IBS and PD remained and constipation could not entirely explain such association (32).

It is important to determine the causal relationship between IBS and PD. Except for the study conducted by Lee et al. (33), all PD patients were diagnosed after the IBS onset, which seems to attempt to demonstrate that IBS is a risk factor for PD (26, 31, 32). However, non-motor symptoms of PD often occur before the onset of motor symptoms, so IBS may be a manifestation of early PD that has not been diagnosed. In the Finnish study, the HR for PD in subjects with IBS compared to those without IBS was highest during the first 2 years of follow-up and the association decreased between 2 and 5 years of follow-up, which appeared to support the reverse causation (26). However, the HR increased after more than 5 years of follow-up, although statistical significance was not observed, possibly due to reduced statistical power limited by a smaller sample size. When the definition for PD was more liberal to include more samples, statistical significance again occurred (26). The association between IBS and elevated PD risk was still observed in patients followed for more than 2 years in the Taiwanese population (31). Furthermore, the Swedish study showed a 38% increased risk of PD among subjects with IBS diagnosis for more than 10 years (32). Therefore, the current evidence did not completely explain IBS as a premotor symptom of PD. In addition, the association between IBS and PD may be the result of a common exposure.

In the included studies, except for the small sample study conducted by Lee et al. (33), the results of three nationwide or regional studies adjusted for a variety of possible confounding factors (26, 31, 32). However, only the Swedish study assessed the impact of surveillance bias on results (32). Subjects with IBS often have a greater number of medical visits than those without IBS, so they are more likely to notice their body changes and have earlier and greater probability of getting the PD diagnosis. Therefore, it is possible to overestimate the increased risk of PD in subjects with IBS during the whole period of analysis. In addition, this may partly explain that subjects with IBS had the highest HR for PD in the first 2 years of follow-up compared to those without IBS. In the Swedish study, the association was attenuated after adjusting the total number of hospital visits, but it remained significant.

The exact mechanism of interaction between IBS and PD remains unclear. Many factors related to IBS, such as low-grade mucosal inflammation, immune activation disorders, altered intestinal permeability, metabolic abnormalities, and disturbances of the neuroendocrine system, may include some of the fundamental elements of PD (19). Furthermore, these factors could make the gastrointestinal tract more vulnerable to pathogens, thereby becoming the initiation site for α-synuclein aggregation (22, 35). The correlation between the bacterial gut disturbances and the development and severity of IBS has been observed in previous studies, and the gut-brain axis plays a central role in the perpetuation of IBS, which are similar to the hypothesis that PD originates in the gut (36–38). When intestinal dysfunction and/or intestinal flora are disturbed, it may cause local and systemic inflammation and enteric neuroglial activation, which will eventually trigger the development of α-synuclein pathology (13, 39). However, further research is needed to confirm the correlation, and reverse causality is possible.

There are some limitations in this study. First, the number of included studies is relatively small, so it is unclear whether this connection exists in other ethnicities and regions in the world. Second, although the population from the national or regional databases provided a large sample for the study. However, the data in these databases are not collected for specific research questions, so some potential confounding factors that may affect the risk of PD, such as Body Mass Index, dietary habits, exposure to pesticides, coffee consumption, and medication use were not controlled. Moreover, it is unclear whether there are differences in the association between the different subtypes of IBS and PD. Next, the sensitivity and specificity of using diagnostic codes to determine patients are limited, and underdiagnosis and misclassification of IBS and PD are inevitable. Fifth, there is a time error in the diagnosis of IBS and PD, so the temporal relationship analysis may not be accurate. Finally, although Egger' and Begg' tests showed no publication bias, there may still be potential publication bias due to the small number of studies.

## Conclusion

The present meta-analysis indicated that there is a significant association between IBS and PD, and subjects with IBS have a higher risk of developing PD compared to those without IBS. This increased risk was observed in both genders and increased with the age at IBS diagnosis. However, it is unclear whether IBS is a risk factor for PD, and there may be a reverse causation. Future studies with large samples and long follow-up in different geographic areas are required to further confirm this association and clarify the causation and pathological mechanism between IBS and PD.

## Data Availability Statement

The original contributions generated for the study are included in the article/Supplementary Material, further inquiries can be directed to the corresponding author/s.

## Author Contributions

QS and ZS contributed to the conception and design of the study. XZ and QH searched the database. XZ, YY, and ML extracted data. ZS and RZ conducted data analysis. XZ, ZS, and ML wrote and revised the manuscript. All authors contributed to manuscript revision, read, and approved the submitted version.

## Funding

This study was supported by the International Cooperation Project on new Traditional Chinese medicine for prevention and treatment of Parkinson's disease (00023129) and the project of Sichuan Administration of traditional Chinese medicine, 2021MS107 [czybf (2021) No. 13-130].

## Conflict of Interest

The authors declare that the research was conducted in the absence of any commercial or financial relationships that could be construed as a potential conflict of interest.

## Publisher's Note

All claims expressed in this article are solely those of the authors and do not necessarily represent those of their affiliated organizations, or those of the publisher, the editors and the reviewers. Any product that may be evaluated in this article, or claim that may be made by its manufacturer, is not guaranteed or endorsed by the publisher.
