# Supplementary material for: Association Between Irritable Bowel Syndrome and Risk of Parkinson's Disease: A Systematic Review and Meta-Analysis
Source: Front Neurol. 2021 Sep 22;12:720958. doi: 10.3389/fneur.2021.720958 (PMC8492947; doi:10.3389/fneur.2021.720958)
Supplement: Supplementary file 1 [file Data_Sheet_1.DOCX]

**Search strategy of each database:**

**PubMed:**

((irritable bowel syndromes or Syndrome, Irritable Bowel or Syndromes, Irritable Bowel or Colon, Irritable or Irritable Colon or Colitis, Mucous or Colitides, Mucous or Mucous Colitides or Mucous Colitis) OR ("Irritable Bowel Syndrome"[Mesh])) AND ((Idiopathic Parkinson's Disease or Lewy Body Parkinson's Disease or Parkinson's Disease, Idiopathic or Parkinson's Disease, Lewy Body or Parkinson Disease, Idiopathic or Parkinson's Disease or Idiopathic Parkinson Disease or Lewy Body Parkinson Disease or Primary Parkinsonism or Parkinsonism, Primary or Paralysis Agitans) OR ("Parkinson Disease"[Mesh]))

**Web of Science:**

TS=(colon spasm or colon, irritable or colonic diseases, functional or colonospasm or functional colonic diseases or irritable bowel syndrome or irritable colon syndrome or mucomembraneous colitis or mucomembranous colitis or mucous colitis or spastic colitis or spastic colon or unstable colon or irritable colon) OR (idiopathic parkinsonism or Lewy bodies of Parkinson disease or Lewy bodies of Parkinson's disease or Lewy bodies of Parkinsons disease or Lewy body Parkinson disease or Lewy body Parkinson's disease or Lewy body Parkinsons disease or paralysis agitans or Parkinson dementia complex or Parkinson's disease or Parkinsons disease or primary parkinsonism or Parkinson disease)

Databases: SCI-EXPANDED, SSCI, A&HCI, CPCI-S, CPCI-SSH, BKCI-S, BKCI-SSH, ESCI, CCR-EXPANDED, IC Timespan=All years

**Embase:**

Sources: Embase, MEDLINE

Query ('parkinson disease'/exp OR 'idiopathic parkinsonism':ab,ti OR 'lewy bodies of parkinson disease':ab,ti OR 'lewy bodies of parkinsons disease':ab,ti OR 'lewy body parkinson disease':ab,ti OR 'lewy body parkinsons disease':ab,ti OR 'paralysis agitans':ab,ti OR 'parkinson dementia complex':ab,ti OR 'parkinsons disease':ab,ti OR 'primary parkinsonism':ab,ti) AND ('irritable colon'/exp OR 'colon spasm':ab,ti OR 'colon, irritable':ab,ti OR 'colonic diseases, functional':ab,ti OR 'colonospasm':ab,ti OR 'functional colonic diseases':ab,ti OR 'irritable bowel syndrome':ab,ti OR 'irritable colon syndrome':ab,ti OR 'mucomembraneous colitis':ab,ti OR 'mucomembranous colitis':ab,ti OR 'mucous colitis':ab,ti OR 'spastic colitis':ab,ti OR 'spastic colon':ab,ti OR 'unstable colon')

**Cochrane library:**

ID        Search

#1        MeSH descriptor: [Irritable Bowel Syndrome] explode all trees

#2        Colitides, Mucous or Colon, Irritable or Syndrome, Irritable Bowel or Syndromes, Irritable Bowel or Colitis, Mucous or Mucous Colitis or Irritable Colon or Mucous Colitides or Irritable Bowel Syndromes

#3        MeSH descriptor: [Parkinson Disease] explode all trees

#4        Idiopathic Parkinson's Disease or Parkinson's Disease, Lewy Body or Primary Parkinsonism or Parkinsonism, Primary or Parkinson Disease, Idiopathic or Lewy Body Parkinson's Disease or Parkinson's Disease or Paralysis Agitans or Idiopathic Parkinson Disease or Parkinson's Disease, Idiopathic or Lewy Body Parkinson Disease

#5        #1 or #2

#6        #3 or #4

#7        #5 and #6

**The list of articles excluded after a full-text reading**

1. Mertsalmi TH, Aho VTE, Pereira PAB, Paulin L, Pekkonen E, Auvinen P, Scheperjans F. More than constipation - bowel symptoms in Parkinson's disease and their connection to gut microbiota. Eur J Neurol. 2017 Nov;24(11):1375-1383. doi: 10.1111/ene.13398.
2. Mertsalmi T, Pekkonen E, Scheperjans F. Diagnosis of irritable bowel syndrome increases risk of Parkinson's disease in the Finnish population. Movement Disorders 2017 32 Supplement 2 (54 - 55). doi: 10.1002/mds.27087
3. Acar BA, Acar MAG, Acar T, Varım C, Alagöz AN, Demiryürek EB, Doğan Güngen B, Güzey Aras Y. Patients with primary restless legs syndrome have higher prevalence of autonomic dysfunction and irritable bowel syndrome. Singapore Med J. 2018 Oct;59(10):539-544. doi: 10.11622/smedj.2018010.
4. Mishima T, Fukae J, Fujioka S, Inoue K, Tsuboi Y. The Prevalence of Constipation and Irritable Bowel Syndrome in Parkinson's Disease Patients According to Rome III Diagnostic Criteria. J Parkinsons Dis. 2017;7(2):353-357. doi: 10.3233/JPD-160982.
5. Kachru N, Holmes HM, Johnson ML, Chen H, Aparasu RR. Antimuscarinic use among older adults with dementia and overactive bladder: a Medicare beneficiaries study. Curr Med Res Opin. 2021 Aug;37(8):1303-1313. doi: 10.1080/03007995.2021.1920899.
6. Mishima T, Fukae J, Fujioka S, Inoue K, Tsuboi Y. The prevalence of constipation and irritable bowel syndrome in Parkinson's disease patients according to Rome III diagnostic criteria. Movement Disorders 2016 31 Supplement 2 (S111-S112). doi: 10.1002/mds.26688
7. Shah E, Pimentel M. Placebo effect in clinical trial design for irritable bowel syndrome. J Neurogastroenterol Motil. 2014 Apr 30;20(2):163-70. doi: 10.5056/jnm.
8. Zhu S, Wang Z, Duan L. Genetic associations of irritable bowel syndrome and depressive disorder through whole exome pooled-sequencing. United European Gastroenterology Journal. 2018 6:8 Supplement (A63-A64). doi: 10.1177/2050640618792817
9. Resnikoff H, Metzger JM, Lopez M, Bondarenko V, Mejia A, Simmons HA, Emborg ME. Colonic inflammation affects myenteric alpha-synuclein in nonhuman primates. J Inflamm Res. 2019 May 7;12:113-126. doi: 10.2147/JIR.S196552.
10. Clairembault T, Leclair-Visonneau L, Coron E, Bourreille A, Le Dily S, Vavasseur F, Heymann MF, Neunlist M, Derkinderen P. Structural alterations of the intestinal epithelial barrier in Parkinson's disease. Acta Neuropathol Commun. 2015 Mar 10;3:12. doi: 10.1186/s40478-015-0196-0.
11. Zhu S.-W, Liu Z.-J, Wei H, Duan L.-P. Genome-wide associations for irritable bowel syndrome through meta-analysis and whole exome pooled-sequencing. Journal of Digestive Diseases 2018 19 Supplement 1 (128-129). doi: 10.1111/1751-2980.12665
12. Julio-Pieper M, O'Connor R.M, Dinan T.G, Cryan J.F. Regulation of the brain-gut axis by group III metabotropic glutamate receptors. European Journal of Pharmacology 2013 698:1-3 (19-30). doi: 10.1016/j.ejphar.2012.10.027
13. Dinan TG, Cryan JF. The Microbiome-Gut-Brain Axis in Health and Disease. Gastroenterol Clin North Am. 2017 Mar;46(1):77-89. doi: 10.1016/j.gtc.2016.09.007.
14. Ghaisas S, Maher J, Kanthasamy A. Gut microbiome in health and disease: Linking the microbiome-gut-brain axis and environmental factors in the pathogenesis of systemic and neurodegenerative diseases. Pharmacol Ther. 2016 Feb;158:52-62. doi: 10.1016/j.pharmthera.
15. Cirstea M, Golz E, MacVicar B, Finlay B, Cresswell S. The Gut Microbiota in a Canadian Parkinson's Disease Cohort. Movement Disorders 2018 33 Supplement 2 (S716-)
16. Bell IR, Amend D, Kaszniak AW, Schwartz GE, Peterson JM, Stini WA, Miller JW, Selhub J. Trait shyness in the elderly: evidence for an association with Parkinson's disease in family members and biochemical correlates. J Geriatr Psychiatry Neurol. 1995 Jan;8(1):16-22.
17. Mischley L. Tips and tricks for living with Parkinson's that go beyond medication - Nutrition and constipation. Journal of Parkinson's Disease 2019 9:1 (4-). doi: 10.3233/JPD-199900
18. Evrensel A, Ceylan M.E. Fecal microbiota transplantation and its usage in neuropsychiatric disorders. Clinical Psychopharmacology and Neuroscience 2016 14:3 (231-237). doi: 10.9758/cpn.2016.14.3.231
19. Mertsalmi T, Aho V, Pereira P.A.B, Paulin L, Pekkonen E, Auvinen P, Scheperjans F. Irritable bowel syndrome is more prevalent than functional constipation in Parkinson's disease: Clinical spectrum and changes in gut microbiota. Movement Disorders 2016 31 Supplement 2 (S111-S112). doi: 10.1002/mds.26688
20. Murtomäki K, Mertsalmi T, Jaakkola E, Mäkinen E, Joutsa J, Levo R, Pekkonen E, Kaasinen V, Scheperjans F. Functional bowel symptoms in Parkinson's disease. Movement Disorder 2019 34 Supplement 2 (S655-)
